# The protective role of intracellular glutathione in *Saccharomyces cerevisiae* during lignocellulosic ethanol production

**DOI:** 10.1186/s13568-020-01148-7

**Published:** 2020-12-17

**Authors:** Vijayendran Raghavendran, Christian Marx, Lisbeth Olsson, Maurizio Bettiga

**Affiliations:** 1 Department of Biology and Biological Engineering, Chalmers University of Technology, Kemivägen 10, 412 96 Göteborg, Sweden; 2 Present Address: Faculty of Sciences I-Biosciences, Institute of Pharmacy, Department of Pharmaceutical Technology and Biopharmacy, Martin-Luther-Universität Halle-Wittenberg, Weinbergweg 22, 06120 Halle (Saale), Germany; 3 EviKrets Biobased Processes Consultants, Lunnavägen 87, 438 34 Landvetter, Sweden

**Keywords:** Bioethanol, Fermentation, Glutathione, Lignocellulosic inhibitors, SSF, Toxicity

## Abstract

To enhance the competitiveness of industrial lignocellulose ethanol production, robust enzymes and cell factories are vital. Lignocellulose derived streams contain a cocktail of inhibitors that drain the cell of its redox power and ATP, leading to a decrease in overall ethanol productivity. Many studies have attempted to address this issue, and we have shown that increasing the glutathione (GSH) content in yeasts confers tolerance towards lignocellulose inhibitors, subsequently increasing the ethanol titres. However, GSH levels in yeast are limited by feedback inhibition of GSH biosynthesis. Multidomain and dual functional enzymes exist in several bacterial genera and they catalyse the GSH biosynthesis in a single step without the feedback inhibition. To test if even higher intracellular glutathione levels could be achieved and if this might lead to increased tolerance, we overexpressed the genes from two bacterial genera and assessed the recombinants in simultaneous saccharification and fermentation (SSF) with steam pretreated spruce hydrolysate containing 10% solids. Although overexpressing the heterologous genes led to a sixfold increase in maximum glutathione content (18 µmol g_drycellmass_^−1^) compared to the control strain, this only led to a threefold increase in final ethanol titres (8.5 g L^− 1^). As our work does not conclusively indicate the cause-effect of increased GSH levels towards ethanol titres, we cautiously conclude that there is a limit to cellular fitness that could be accomplished via increased levels of glutathione.

## Introduction

Targeting industrial chemicals with bio-based processes is an emerging market as it enables the production from non-petrochemical feedstock. Biomass is omnipresent but the complex crystalline lignocellulose matrix is highly recalcitrant by nature (McCann and Carpita 2015). Pre-treatment aids in reducing the crystallinity so that the carbohydrate degrading enzymes can access cellulose and hemicellulose and break them down into soluble sugars. Inevitably, most of the pretreatment methods currently available (Kumar and Sharma 2017) produce inhibitors (e.g. aromatic aldehydes, organic acids) (Jönsson et al. 2013; Taherzadeh 1999) that adversely affect the efficiency of microorganisms or the saccharifying enzymes both of which have implications on the final product cost.

Numerous strategies have been reported for overcoming the challenges posed by lignocellulosic inhibitors to increase the yield and productivity of ethanol (Jönsson and Martín 2016; Kim 2018; Kumar et al. 2020; Wang et al. 2018). We focused our approach on glutathione (GSH), the cellular protectant that plays a major role in detoxifying reactive oxygen species and free radicals (Grant 2001; Meister and Anderson 1983). Upon oxidative stress, GSH is oxidised to Glutathione disulfide GSSG by reactions with free radicals. Yeast strains lacking GSH are sensitive to oxidative stress (Izawa et al. 1995). Furfural and hydroxy methyl furfural, inhibitors that are present in lignocellulosic hydrolysate act as thiol reactive electrophiles depleting GSH levels in *Saccharomyces cerevisiae*. Increasing GSH levels by increasing the expression levels of genes (*GSH1* and *GLR1)* in the GSH biosynthetic pathway or the exogenous addition of GSH led to tolerance towards furfural (Kim and Hahn 2013).

**Fig. 1:**
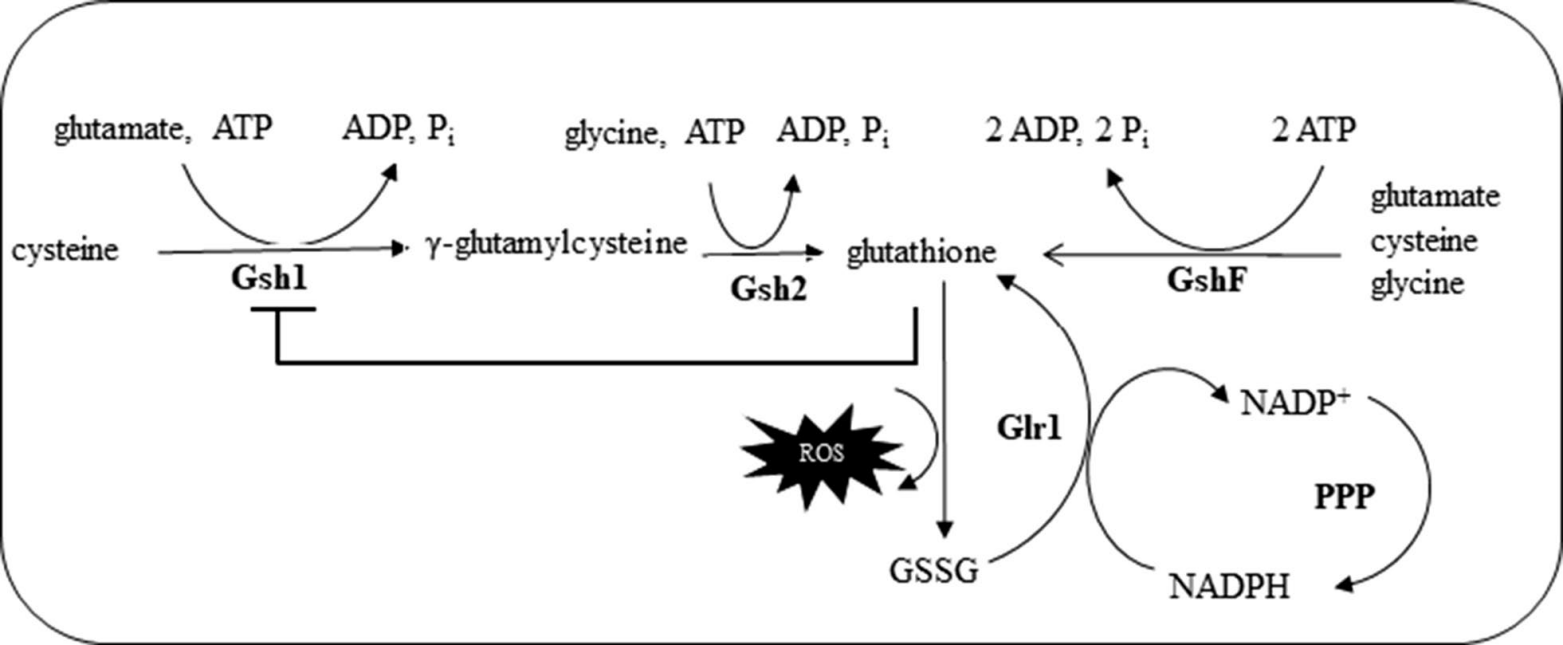
Glutathione biosynthesis in yeast is a two-step process catalysed by Gsh1 and Gsh2 involving three amino acids: cysteine, glutamate, glycine, and two molecules of ATP. In some bacteria, the bioconversion is carried out in a single-step pathway by a bifunctional enzyme GshF that is free from feedback inhibition of the product, unlike in yeast where GSH inhibits the activity of Gsh1. During deactivation of reactive oxygen species (ROS), GSH is oxidised to GSSG. Oxidised GSSG is reduced back to GSH by glutathione reductase (Glr1) using NADPH as a co-factor. In actively growing cells, the pentose phosphate pathway (PPP) supplies the NADPH

Previously, we overexpressed the genes in the GSH biosynthetic pathway in *S. cerevisiae* and observed a concomitant increase in the yield of ethanol (Ask 2013). In yeast, glutathione biosynthesis is a two-step process (Fig. 1). The first enzyme is feedback inhibited by high levels of glutathione. In our quest for enzymes that were free from feedback inhibition and that would enable even further accumulation of glutathione, we identified two bacterial single step multicatalytic enzymes from *Streptococcus thermophilus* (Li et al. 2011) and *Listeria monocytogenes* (Gopal et al. 2005). In the present study, we investigated the effect of increasing the intracellular glutathione content in yeast. We then assessed the performance of the engineered strains during simultaneous saccharification and fermentation (SSF) using steam pretreated spruce hydrolysate. We report that ethanol titres increased linearly with increasing glutathione content. However, cellular fitness reached an upper limit during lignocellulosic ethanol fermentation in harsher conditions (10% solids) indicating that an increased glutathione content only partly counteracts the inhibitory effect of hydrolysates.

## Materials and methods

### Microorganisms

*Saccharomyces cerevisiae (Sc)* strains employed in the study were derived from CEN.PK 113-5D *(Mat a ura3-52 HIS3 LEU2 TRP1 MAL2-8c SUC2).* It was made prototrophic by integrating the plasmid YIpLac211 in the *ura3* locus and this constitutes the control strain. The same vector was used to create the recombinant strains. The plasmid, harbouring the gene for overexpression was integrated in the *ura3* locus of CEN.PK 113-5D strain, resulting in prototrophic strains overexpressing the relevant glutathione biosynthetic pathway genes. The list of strains used in this study is shown in Table 1.

The coding region of *Streptococcus thermophilus* (*St*) GSH fusion enzyme StGCS-GS (GenBank accession no. GQ848551) and *Listeria monocytogenes* (*Lm*; EGD-e: lmo2770) were ordered as gene synthesis from GenScript USA Inc. (Piscataway, NJ, USA) and received them subcloned into pUC57. They were subsequently transferred to YIpLac211 to yield the constructs shown in Table 1.

**Table 1:** List of microorganisms employed in this study

Strain	Strain genotype
Reference strain	*MATa MAL2-8*^*c*^ *SUC2 ura3-52::URA3-YIp211*
*GSH1* (Ask et al. 2013b)	*MATa MAL2-8*^*c*^ *SUC2 ura3-52::URA3-TDH3p-GSH1_Sc-Cyc1t*
*LmgshF*	*MATa MAL2-8*^*c*^ *SUC2 ura3-52::URA3-TDH3p-GshF_Lm-Cyc1t*
*StgshF*	*MATa MAL2-8*^*c*^ *SUC2 ura3-52::URA3-TDH3p-GshF_St-Cyc1t*
*Δgsh2 LmgshF*	*MATa MAL2-8*^*c*^ *SUC2 ura3-52::URA3-TDH3p-GshF_Lm-Cyc1t gsh2::loxP::loxP*

Strains were stored in − 80 ^o^C glycerol (30% v/v) + YPD stocks (Yeast extract 10 g L^− 1^, Peptone 20 g L^− 1^ and glucose 20 g L^− 1^). Before every experiment, agar plates were prepared by streaking the culture onto a YPDA plate (Yeast extract 10 g L^− 1^, Peptone 20 g L^− 1^ and glucose 20 g L^− 1^, agar 20 g L^− 1^) and incubated at 30 °C for 48 h, to obtain single colonies

### Shake flask cultivation

The medium for preinoculum was made according to Verduyn (Verduyn et al. 1992) but with 50 mM potassium phthalate buffer (pH 5) to maintain culture pH during the cultivation. A single colony was transferred to 2 mL of defined medium in a culture tube, at 30 °C and 200 rpm, for 24 h. For growth curve experiments, 250 mL cotton stoppered Erlenmeyer flasks containing 50 mL of medium were inoculated with preinoculum cultures to reach an initial absorbance (600 nm) of 0.2. Samples were taken every hour during the exponential phase for absorbance measurements.

### Calculation of physiological parameters

The maximum specific growth rate was obtained by plotting the natural logarithm of absorbance against time in the exponential growth phase. The slope of the straight line obtained by linear regression represented the *µ*_*max*_.

### Simultaneous Saccharification and Fermentation

SSF was carried out as described by Ask et al. (2013b). Briefly, cotton stoppered shake-flasks were used with a working mass of 50 g containing water insoluble solids (WIS) content of 5 or 10% (w/w) steam-pretreated spruce (SEKAB E-Technology Örnsköldsvik, Sweden). The pH of the slurry was adjusted to 5 with 3 M KOH and supplemented with 1 g L^− 1^ yeast extract, 0.5 g L^− 1^ (NH_4_)_2_HPO_4_, 0.025 g L^− 1^ MgSO_4_·7H_2_O and 50 mM citrate buffer at pH 5. The SSF experiment began by addition of cell suspension yielding a final dry cell biomass concentration of 4 g·L^− 1^ and Cellic CTec2 (Novozymes A/S, Bagsvaerd, Denmark) at 20 mg enzyme preparation per g_solids_ (corresponding to ca. 3 FPU), at 35 °C and 150 rpm. Samples were withdrawn regularly throughout the cultivations that were performed in duplicate, centrifuged and the supernatants were filtered through 0.2 µm nylon syringe-filters and stored at − 20 °C until further analysis.

### Analytical determinations

Glucose and ethanol were determined by an HPLC using the Rezex column at 80 °C with an RI detector, at a flow of 0.8 mL min^− 1^ of 5 mM H_2_SO_4_. The intracellular GSH was determined during early exponential phase as described by our earlier publication (Ask et al. 2013b) using the 5, 5′-dithiobis-(2-nitrobenzoic acid) method (Morgan et al. 2012; Rahman et al. 2006). Normalised GSH values were obtained with an absorbance-dry mass correlation of the strains employed (g dry cell mass per OD for various strains: Reference strain 0.53; *GSH1* 0.55; *LmgshF* 0.54; *StgshF* 0.47; *Δgsh2 LmgshF* 0.525).

## Results

### Recombinant strains have increased GSH levels without compromising growth

Strains harbouring alternative enhanced pathways for glutathione were obtained in the *S. cerevisiae* CEN.PK background. We overexpressed separately the endogenous gene in the first step of the GSH biosynthetic pathway, as well as the gene encoding the bacterial single step multicatalytic enzyme *LmgshF* and *StgshF*, under the control of a strong constitutive promoter (*TDH3*). We also overexpressed the *LmgshF* in a *Δgsh2* background. We used a prototrophic derivative of the parent strain obtained by integrating the auxotrophic marker into the genome, as the reference strain. The obtained strains are designated *GSH1, LmgshF, StgshF*, and *Δgsh2 LmgshF*, respectively.

When introducing any genetic changes, it is vital to see their effect on microbial growth. Thus, we determined the maximum specific growth rate (*µ*_*max*_, h^− 1^) of the strains (Table 2) under investigation in a defined mineral medium (Verduyn et al. 1992). Overexpression of either the endogenous gene or the bacterial genes (*LmgshF* or *StgshF*) did not affect the maximum specific growth rate significantly. The maximum specific growth rate of all the strains was. 0.40–0.42 h^− 1^.

To assess the effect of overexpression of the genes in various strain backgrounds, the reduced and oxidised intracellular glutathione levels were measured during the early exponential phase in a defined mineral medium (Table 2). Compared to the reference strain, *GSH1* overexpression led to a twofold increase in the reduced glutathione levels and a sixfold increase for *LmgshF, StgshF*, and *Δgsh2 LmgshF*. The oxidised GSH were threefold higher for the recombinant strains. Cellular redox is determined not only by the absolute concentration of the reduced GSH but also by the GSH : GSSG ratio. The ratio (Table 2) was 88–95 for *LmgshF, StgshF* and *Δgsh2 LmgshF* but it was lower than the reference strain for *GSH1* at 32.

**Table 2:** Maximum specific growth rate and intracellular concentrations of reduced and oxidized glutathione in the strains used in this study in a defined mineral medium

Strain	*µ*_*max*_ (h^− 1^)	GSH µmol g_drycellmass_^−1^	GSSG µmol g_drycellmass_^−1^	[GSH]:[GSSG] ratio
Reference strain	0.40 ± 0.01	3.1 ± 0.5	0.07 ± 0.02	45 ± 16
*GSH1*	0.40 ± 0.00	6.6 ± 0.4	0.21 ± 0.00	32 ± 2
*LmgshF*	0.42 ± 0.01	17.3 ± 0.4	0.20 ± 0.01	88 ± 6
*StgshF*	0.42 ± 0.00	18.6 ± 0.1	0.22 ± 0.03	86 ± 12
*Δgsh2 LmgshF*	0.40 ± 0.01	18.8 ± 1.0	0.20 ± 0.00	95 ± 5

Data represent the mean ± standard deviation (n = 2). Total GSH is the sum of GSH and GSSG. Ask et al. (2013b) overexpressed *GSH1* in a prototrophic CEN.PK 113-7D background and reported a GSH content of 12.2 µmol g_drycellmass_^−1^

### Recombinant strains perform as well as the reference strain during SSF with 5% solids

**Fig. 2:**
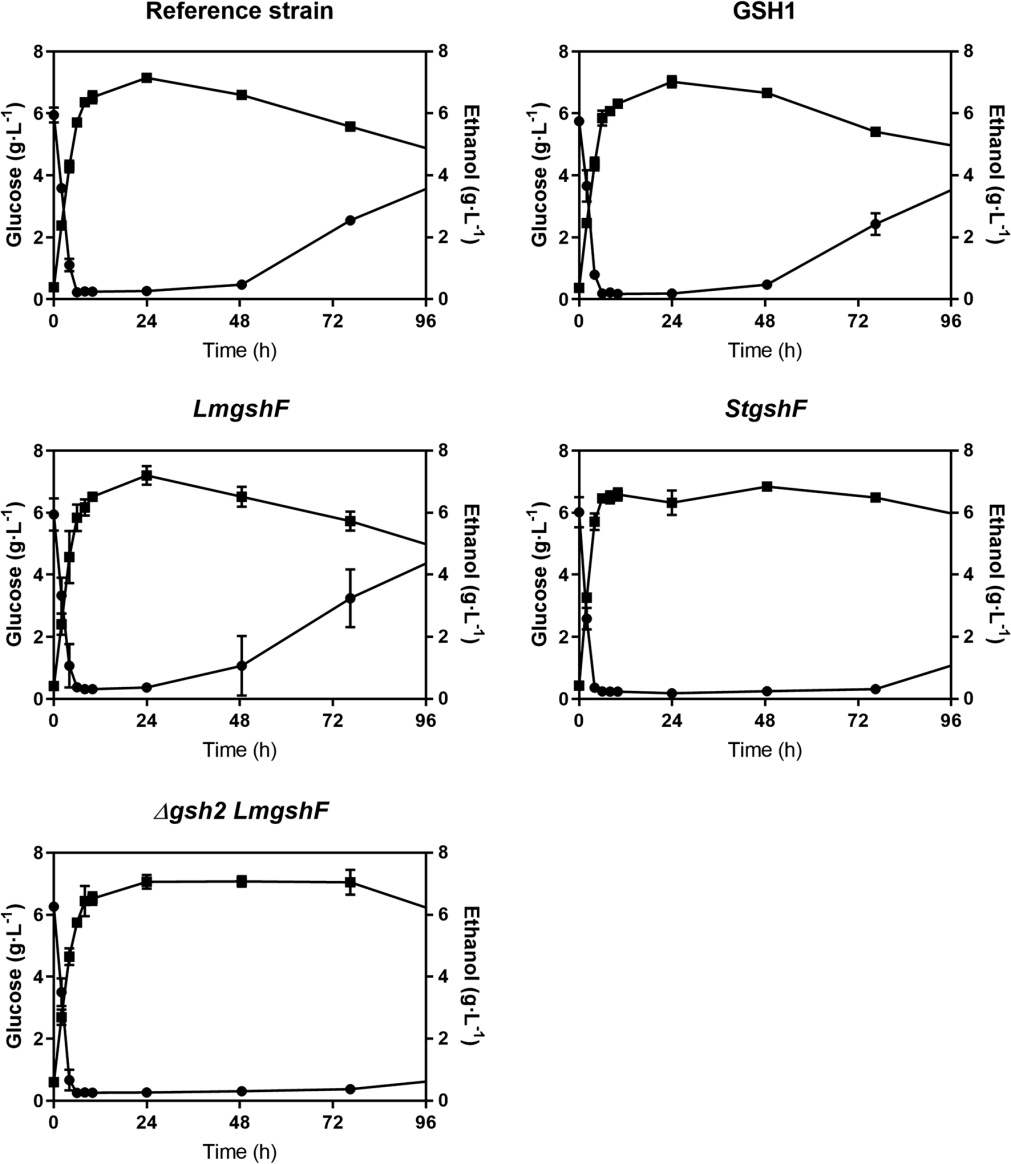
The time course of glucose (filled circles) and ethanol (filled squares) concentrations with the *Saccharomyces cerevisiae* strains investigated in the present study with spruce hydrolysate containing 5% WIS content and an initial dry cell biomass concentration of 4 g L^− 1^ using 20 mg enzyme preparation per g_solids_ at 35 °C and 150 rpm. The data are the means and standard deviation (n = 2)

To assess if the increased glutathione content observed (see above) translate into increased ethanol titres, the recombinant strains were subjected to SSF with steam treated spruce hydrolysate. We used a temperature of 35 °C as a compromise between yeast growth and enzyme optima and a WIS content of 5%, implying an inhibitor concentration that is likely to be tolerated by all the strains. In fact, all the strains exhibited comparable initial performance, with similar ethanol production up to 10 h (Fig. 2). After 10 h, all the free glucose was exhausted, and the process was only limited by the saccharification rate. Only after 48 h, sugar accumulation was observed reaching a maximum of 4.5 g L^− 1^ for *LmgshF*, followed by 3.9 g L^− 1^ for *GSH1* and the reference strain. The lowest glucose concentrations were 1.2 and 0.7 g L^− 1^ for *StgshF* and *Δgsh2 LmgshF* respectively, indicating that these strains could sustain glucose consumption for a longer time.

### Recombinant strains exhibited a differential response during SSF with 10% solids

**Fig. 3:**
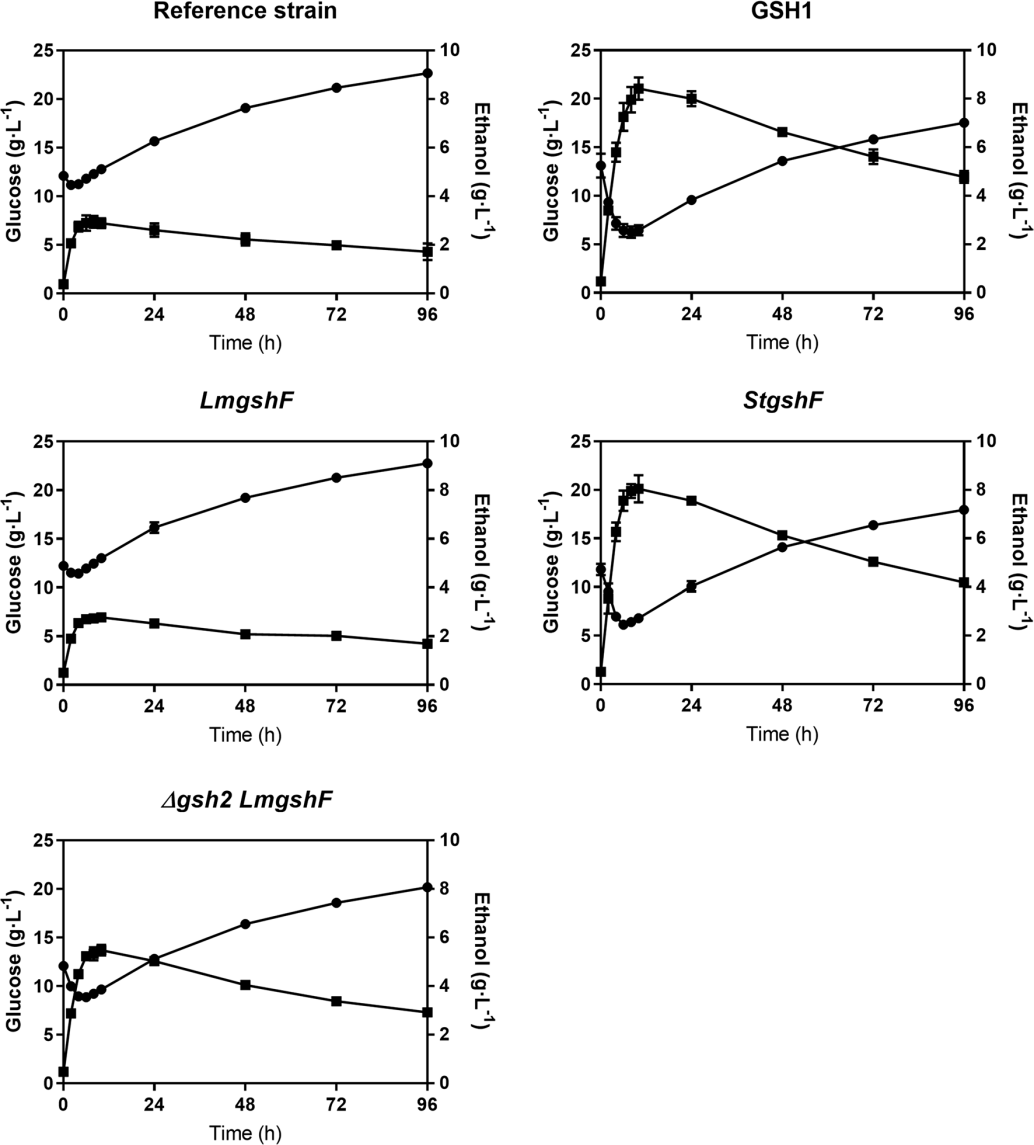
The time course of glucose (filled circles) and ethanol (filled squares) concentrations with the *Saccharomyces cerevisiae* strains investigated in the present study with spruce hydrolysate containing 10% WIS content and using the same operating conditions as described in Fig. 3. The data are the means and standard deviation (n = 2)

As there were no discernible differences in the physiology of the recombinant strains during SSF using 5% solids, the WIS content was increased to 10% under the hypothesis that a higher concentration of inhibitors present in the raw material would enable us to delineate the phenotypic differences between the strains without killing the cells rapidly. All the recombinant strains except the *LmgshF* strain exhibited superior performance compared to the reference strain (Fig. 3). All strains started to consume glucose instantaneously after inoculation (Fig. 3). Glucose began to accumulate after 4 h for *LmgshF* and the reference strain, after 6 h for *Δgsh2 LmgshF* and after 8 h for *GSH1* and *StgshF*. The highest ethanol titres were achieved for *GSH1* and *StgshF* (8.0 and 8.4 g L^− 1^), followed by *Δgsh2 LmgshF* at 5.5 g L^− 1^. The lowest ethanol titre was obtained with the reference strain and *LmgshF*.

### The correlation between GSH content and fermentation performance is nonlinear .

**Fig. 4:**
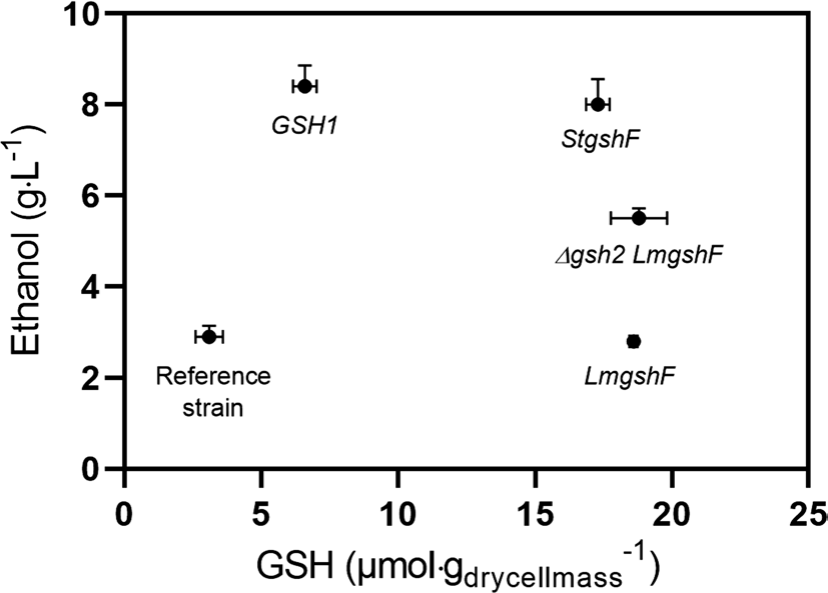
Correlation between intracellular GSH levels measured in a defined mineral medium and the ethanol titres obtained during SSF with 10% WIS. The data are the means and standard deviation (n = 2)

To verify if there is a relationship between the ethanol titres observed with 10% SSF and the glutathione content, the glutathione levels measured during the early exponential phase were plotted against the ethanol titres (Fig. 4). As the glutathione content increased three and sixfold, the ethanol titre increased only threefold, and any further increase in glutathione did not result in increased titres.

## Discussion

The lignocellulosic hydrolysate is a motley mixture of various chemical compounds that exert significant stress on cells, leading to decreased overall productivity. Tolerating stress is vital for developing robust industrial strains (Attfield 1997; Stelling et al. 2004). GSH is a key antioxidant in eukaryotic cells and it has a plethora of functions (Meister and Anderson 1983; Penninckx 2002) including detoxifying the xenobiotic compounds, activating virulence (Reniere et al. 2015) and conferring fitness to hawkmoths during flight (Levin et al. 2017). As the native Gsh1p is feedback inhibited by GSH, we cloned and expressed separately two bacterial multicatalytic enzymes in yeast. The overexpression of the genes had no effect on the specific growth rate when grown in a synthetic defined medium.

Qiu and co-workers overexpressed the *StgshF* gene in the BY 4717 yeast strain and reported a GSH level of 56 µmol·g_drycellmass_^−1^ (Qiu et al. 2015) in a YPD medium, corresponding to an intracellular concentration of 24 mM. As the YPD supplied the three amino acids necessary for GSH biosynthesis, a higher value for GSH accumulation is expected. Ge and co-workers overexpressed *LmgshF* in *Pichia pastoris* and reported a value of 40 µmol g_drycellmass_^−1^ also in YPD medium (Ge et al. 2012). These data, though in the same order of magnitude, are significantly higher than the one achieved by the recombinant strains, *LmgshF* and *StgshF.* However, it has to be considered that in the present study GSH measurements were performed on strains grown in defined mineral medium; in fact, when we supplemented our defined medium with the precursor amino acids, we also achieved much higher GSH levels although the maximum specific growth rate was reduced (data not shown).

We assessed the performance in an industry-like setting using steam treated spruce hydrolysates at a WIS content of 10%. Three distinct ethanol titres were observed: the reference strain and the *LmgshF* at 2.0 g L^− 1^, the *Δgsh2 LmgshF a*t 5.5 g L^− 1^ and *StgshF* and *GSH1* at 8.5 g L^− 1^. In our previous work, (Ask et al. 2013b) we also observed a twofold increase in ethanol titres for *GSH1* compared to the control strain. Furthermore, to our best knowledge, we report for the first time that the *GSH2* deletion is rescued by complementation with *LmgshF*, as the lack of *GSH2* causes slow growth (Grant et al. 1997), while it is restored in *Δgsh2 LmgshF*. Contrarily, *LmgshF* overexpression has a little effect on the *GSH1* deletion strain, as we observed a 70% reduction in the maximum specific growth rate (data not shown), thereby enunciating the importance of Gsh1p for cell homeostasis and viability besides its function that can be immediately associated with GSH antioxidant function (Lewinska et al. 2014; Tello-Padilla et al. 2018).

GSH is involved in detoxification mechanisms of inhibitors such as hydroxymethyl furfural and furfural (Ask et al. 2013a, b; Qiu et al. 2015). When the intracellular GSH levels were measured (in a synthetic medium in the absence of inhibitors), the recombinants had a threefold (*GSH1*) and a sixfold (*LmgshF, StgshF*, *Δgsh2 LmgshF*) higher level compared to the reference strain while the ethanol levels did not increase proportionately. We anticipated that the ethanol titres would increase proportionately with the GSH levels but the results showed otherwise. In our study, GSH measurements were done in a defined medium during the early exponential phase, while the titres were from SSF experiments done with the hydrolysates. Due to the harsh environment and nutrient constraints encountered under SSF of lignocellulosic slurries, growth and intracellular biosynthetic processes can be assumed to be close to zero. Thus, the cells relied on the accumulated GSH prior to SSF to relieve the toxicity. GSH levels measured during the SSF process at various timepoints would have been better but conducting such an experiment would not be feasible as it is impossible to sample cells from the slurry. We used the ethanol concentration as a proxy of cellular fitness and robustness. During SSF, cell growth is very limited as glycolytic flux supplies them with the ATP needed to remain viable. Stressed cells may produce more ethanol as they need more ATP to expunge the inhibitors or the protons that are accumulating due to the influx of un-dissociated acetic acid. As the ethanol titre did not increase *ad infinitum* when the GSH levels were high, we conclude with caution, that at high glutathione biosynthetic flux, the fitness benefit of the cell might be counterbalanced by the metabolic burden or excessive cofactor drainage by the heterologous pathway itself. Redox homeostasis is regulated by several systems such as NAD^+^/NADH, NADP^+^/NADPH, and GSH/GSSG. Thus great care must be taken to relate the redox potential (Schafer and Buettner 2001) calculated based on whole cell lysates with any observed biological phenomenon as the cytosolic GSSG is often overestimated. (Morgan et al. 2012).

Another key observation in our work is that there is a threshold for tolerance as also reported by Pereira et al. (2016). When we conducted the SSF with 5% solids, all the strains yielded near identical ethanol titres, but at 10% solids, differences in ethanol titres clearly manifested.

In conclusion, this study further strengthens the role of GSH as protective metabolite, with the positive effect on cellular robustness partly correlating with its intracellular concentration. Furthermore, as expected, our data indicate the existence of a trade-off between glutathione biosynthetic activity and cellular viability and fitness beyond a certain threshold, suggesting that an optimum should be aimed for in a possible strain engineering strategy.

## Data Availability

All the data are presented in the main paper.
